# Rhophilin rho GTPase binding protein 1-antisense RNA 1 (RHPN1-AS1) promotes ovarian carcinogenesis by sponging microRNA-485-5p and releasing DNA topoisomerase II alpha (*TOP2A*)

**DOI:** 10.1080/21655979.2021.2002494

**Published:** 2021-12-07

**Authors:** Yi Zhou, Jing Li, Xiaoxin Yang, Yu Song, Haigang Li

**Affiliations:** a Hunan Key Laboratory of the Research and Development of Novel Pharmaceutical Preparations, Changsha, Hunan, China; b Academician Workstation, Changsha Medical University, Changsha, Hunan, China; c Department of Obstetrics and Gynecology, Wuhan Third Hospital, Wuhan, Hubei, China

**Keywords:** RHPN1-as1, miR-485-5p, TOP2A, ovarian cancer, proliferation, apoptosis, migration, invasion

## Abstract

Ovarian cancer (OC) is the most common and lethal gynecological cancer worldwide. Long non-coding RNAs (lncRNAs) and sponging microRNAs (miRNAs) serve as key regulators in the biological processes of OC. We sought to evaluate the effect of the RHPN1-AS1-miR-485-5p-DNA topoisomerase II alpha (TOP2A) axis in regulating OC progression. RHPN1-AS1, miR-485-5p, and TOP2A levels in OC tissues and cells were determined by RT-qPCR. The interaction of RHPN1-AS1/miR-485-5p/TOP2A was assessed using luciferase, RNA immunoprecipitation, and RNA pull-down assays. RHPN1-AS1 silencing allowed us to explore its biological function by measuring cell viability, proliferation, migration, invasion, and apoptosis in OC cells. *In vivo* experiments were performed to verify the *in vitro* findings. We found that the RHPN1-AS1 and TOP2A levels were significantly enhanced, whereas the miR-485-5p levels were reduced in OC tissues and cells. RHPN1-AS1 silencing attenuated cell growth, facilitated apoptosis in OC cells, and inhibited tumor growth *in vivo*. Notably, RHPN1-AS1 negatively regulating miR-485-5p promoted the TOP2A expression in OC cells. In conclusion, RHPN1-AS1 sponging miR-485-5p accelerated the progression of OC by elevating TOP2A expression, which makes it a promising target for the treatment of OC patients.

## Introduction

Ovarian cancer (OC) is the most common and lethal gynecological cancer worldwide [1]. Since patients are often diagnosed with OC at a terminal stage, the high rates of metastasis and mortality are threats to OC patients [2,3]. Improved therapeutic methods, including surgery, chemotherapy, and radiotherapy, still lead to a low 5-year survival rate for OC patients (<35%) [4]. Therefore, exploring the molecular mechanisms regulating OC is essential for providing effective targets for the clinical treatment of OC.

Long non-coding RNAs (lncRNAs) > 200 nucleotides in length, play a crucial role in biological processes by regulating gene expression through sponge effects, even without protein-coding ability [5]. Multiple studies have reported that lncRNAs sponging microRNAs (miRNAs) regulate development, immune response, and tumorigenesis in various cancers [6–8]. Growing evidence has indicated that lncRNA RHPN1-AS1 plays important biological roles in different cancers, such as endometrial, colorectal, and gastric cancer [9–11]. RHPN1-AS1 acts as an oncogene that promotes cell growth, migration, and invasion to aggravate cancer metastasis in these cancers [9–11]. RHPN1-AS1 is also involved in the development of OC by enhancing cancer invasiveness and metastasis by upregulating cell proliferation and reducing cell apoptosis [12–14]. However, the potential effects of RHPN1-AS1 in sponging other miRNAs require further examination.

It is widely accepted that miRNAs are sponged by various lncRNAs, and the abnormal expression of miRNAs regulates cell growth and apoptosis in different types of cancers [15–17]. The miR-485-5p is absorbed by various lncRNAs and further regulates cancer genesis in multiple cancers, including cervical cancer, colorectal cancer, and hepatocellular carcinoma [14,18,19]. Evidence suggests that miR-485-5p serves as both a tumor oncogene and a suppressor in OC progression. Yang et al. [20] suggested that miR-485-5p promotes cell growth by inhibiting SRC expression *in vitro* and *in vivo*. However, Xing et al. [21] clarified that miR-485-5p prevents OC cell progression by downregulating PAK4 expression and that this effect was hampered by lncRNA LINC01224. However, whether RHPN1-AS1 exerts an oncogenic effect on miR-485-5p in OC cells remains unknown.

The topoisomerase II A (TOP2A) gene, which regulates DNA replication and cell division, is an isoform of the TOP2 family, which regulates DNA replication and cell division [22]. The overexpression of TOP2A promotes cell growth and regulates the cell cycle in G2/M [23]. Evidence has shown that high levels of TOP2A contribute to diverse human malignancies, including pancreatic, colon, cervical, and gastric cancers [24–27]. The upregulation of TOP2A is also associated with cell growth in OC, which has been identified as a key biomarker for prognosis in OC patients [28–30]. One study demonstrated that TOP2A elevates the tumorigenesis of high-grade serous OC by activating the TGF-beta/Smad pathway [28]. Additional molecular mechanisms in OC require further investigation.

After bioinformatics analysis, we hypothesized the key role of the RHPN1-AS1/miR-485-5p/TOP2A axis in OC. Therefore, we aimed to examine the role of RHPN1-AS1 *in vitro* and *in vivo* and to ascertain the regulatory relationship between RHPN1-AS1, miR-485-5p, and TOP2A. Our study provides an effective and promising therapeutic target for patients with OC.

## Materials and methods

### Bioinformatics analysis

mRNA microarrays, GSE119056 and GSE23392, including OC samples and non-tumor samples, were downloaded from GEO DataSets (https://www.ncbi.nlm.nih.gov/gds/?term=). These two mRNA microarrays were used to screen out differentially expressed genes, with adjusted P < 0.01 and logFC ≥ 1.5. Another database, GEPIA (http://gepia.cancer-pku.cn/index.html), was also used to identify the differentially expressed genes, with adjusted P < 0.01 and logFC ≥ 1.5. The STRING database (https://string-db.org/) was used to construct a protein-protein interaction network for the differentially expressed genes. TargetScan (http://www.targetscan.org/vert_71/) and starBase algorithms (https://starbase.sysu.edu.cn/index.php) were used to predict the targets of TOP2A and RHPN1-AS1, respectively.

### Patient samples, cells, and cell transfection

Thirty-nine cancer specimens and adjacent normal tissues (i.e., more than 5 cm from tumor tissue) were collected from patients with OC in our hospital between May 2019 and June 2020. Informed consent was obtained from each patient, and the study was approved by the ethics committee of Wuhan Third Hospital (approval number: KY2020-023). Table 1 shows the characteristics of the patients. Human ovarian epithelial cells (HOSEpiC) were purchased from BeNa Culture Collection (Cat#: BNCC340096, China). SKOV3 cells were cultured in McCoy’s 5A medium (Gibco, USA) supplemented with 10% fetal bovine serum (FBS; Invitrogen, USA). CaOV3 cells were cultured in Dulbecco’s modified Eagle medium (HyClone, USA) supplemented with 10% FBS. The HOSEpiC and OVCAR3 cell lines were cultured in RPMI-1640 medium (HyClone) supplemented with 10% FBS and 0.01 mg/mL bovine insulin. All cell lines were maintained at 37°C with 5% CO_2_. SiRNA-RHPN1-AS1, miR-485-5p mimics and inhibitor, SiRNA-TOP2A, and their corresponding negative controls (NC), sh-NC or sh-RHPN1-AS1 were obtained from GenePharm (China). OVCAR3 and SKOV3 cells were transfected for 48 h using Lipofectamine 3000 Transfection Reagent (Invitrogen) and subjected to other functional experiments. The sequences of siRNA, mimics, inhibitor, and their corresponding NC are listed in Supplementary Table 1.

**Table 1.** Associations of tissue RHPN1-AS1 clinicopathological characteristics in 39 ovarian cancer samples

Characteristic	N = 39	RHPN1-AS1 expression		P value
		High n = 20	Low n = 19	
**Age (years)**				0.527
≤ 56	18	8	10	
> 56	21	12	9	
**Tumor Size (cm)**				0.200
≥ 2	23	14	9	
< 2	16	6	10	
**FIGO stage**				0.008*
1	6	1	5	
2	7	1	6	
3	22	14	8	
4	4	4	0	
**Baseline neutrophils > 3.9**				0.092
Yes	33	19	14	
No	6	1	5	
**Histological grade**				0.001*
1	13	1	12	
2	18	13	5	
3	8	6	2	
**Histological type**				0.105
Serous	24	15	9	
Non-serous	15	5	10	
**Distant metastasis**				0.010*
Yes	21	15	6	
No	18	5	13	
**Chemotherapy regimens**				0.476
Carbo + Tax	25	11	14	
Carbo monotherapy	11	7	4	
Carbo + Other	3	2	1	

FIGO, International Federation of Gynecology and Obstetrics; Carbo, Carboplatin; Tax, Taxol (Paclitaxel). *, P < 0.05 using Fisher exact test.

### RT-qPCR assay

The RHPN1-AS1 lncRNA and *TOP2A* mRNA were isolated using TRIzol reagent (Cat#: 15,596,018, Thermo, USA). cDNA was synthesized using a PrimeScript First Strand cDNA Synthesis kit (RR037A, Takara, China), and gene expression was detected using SYBR Premix Ex Taq (DRR420A, Takara, China) according to the following thermocycling conditions: 95°C for 30 s, 40 cycles of 95°C for 5 s, and 60°C for 20 s.

The miR-485-5p was extracted using the miRcute miRNA extraction kit (DP501, Tiangen, China), cDNA was synthesized using the miRcute miRNA First Strand cDNA Synthesis kit (KR211, Tiangen, China), and miRNA expression was detected using the miRcute fluorescence quantitative detection kit (FP411, Tiangen, China) according to the following thermocycling conditions: 95°C for 15 min, 40 cycles of 95°C for 20 s, and 60°C for 34 s.

TOP2A and cytoplasmic control expressions were normalized to glyceraldehyde 3-phosphate dehydrogenase (GAPDH) expression, and U6 was used as an internal control for miR-485-5p and nuclear control. Relative expression was analyzed using the 2^−ΔΔCt^ method [31]. Primer sequences are listed in Table 2.

**Table 2.** The sequences of the primers in this study

Primer	Sequences
**RHPN1-AS1**	Forward: 5ʹ-CTAGCCAGGAGGTTTCGC-3ʹ
	Reverse: 5ʹ-TCCGCAACAAGCACACA-3ʹ
**TOP2A**	Forward: 5ʹ-AGGATTCCGCAGTTACGTGG-3ʹ
	Reverse: 5ʹ-CATGTCTGCCGCCCTTAGAA-3ʹ
**miR-485-5p**	Forward: 5ʹ-CCAAGCTTCACCCATTCCTAACAGGAC-3’
	Reverse: 5ʹ-CGGGATCCGTAGGTCAGTTACATGCATC-3’
**GAPDH**	Forward: 5ʹ-GTCTTCACCACCATGGAGAAG-3’
	Reverse: 5ʹ-CAAAGTTGTCATGGATGACCTTGG-3’
**U6**	Forward: 5ʹ-CTCGCTTCGGCAGCACA-3’
	Reverse: 5ʹ-AACGCTTCACGAATTTGCGT-3’

### Nucleic acid isolation assay

The nucleic acid isolation assay was performed according to a previous study [32]. The OVCAR3 and SKOV3 cell layers were digested into single cells. After resuspension, the cells were centrifuged for 5 min at 4°C and 500 × *g*. Both nuclear and cytoplasmic RNAs from cultured OVCAR3 and SKOV3 cells were isolated using the PARIS Kit (AM1921, Life, USA) according to the manufacturer’s instructions. Briefly, cells were incubated with lysis solution on ice for 10 min after washing with PBS. After centrifugation at 500 × *g*, cytoplasmic RNA was extracted from the supernatant, and nuclear RNA was extracted from the nuclear pellet. U6 and GAPDH were detected in isolated RNAs as controls for nuclear and cytoplasmic RNA, respectively. The RHPN1-AS1 levels from the nuclear and cytosolic fractions were measured separately using RT-qPCR with SYBR Premix Ex Taq (RR420A, Takara, China).

### CCK8 assay

The viability of OVCAR3 and SKOV3 cells was detected using the CCK8 kit (Cat#: K1018; APExBIO, China) according to a previous study [33]. Transfected cells (5 × 10^3^ cells/well) were cultured in 96-well plates. Cell viability at 0, 24, 48, 72, and 96 h was detected by adding 10 µL of CCK8 buffer. After 2 h of incubation, OD450 was measured using a multimode plate reader (Thermo Fisher Scientific).

### 5-Ethynyl-2ʹ-deoxyuridine (EdU) assay

The BeyoClick EdU Cell Proliferation Kit (C0078S, Beyotime, China) was used to determine cell proliferation according to a previous study [34]. Approximately 1 × 10^4^ transfected cells were cultured in 6-well plates. After 48 h, EdU was added to each well at a final concentration of 10 µM for 2 h. Then, the cells were fixed using 4% paraformaldehyde at 25°C for 30 min and then permeabilized with 20% Triton X-100 for 15 min. The cells were then treated with labeled azide and incubated in the dark for 30 min. After washing twice, 4ʹ,6-diamidino-2-phenylindole (Sigma, USA) was used for nuclear staining for 10 min in the dark. Finally, five random photographs were taken using a confocal microscope (Olympus, Tokyo, Japan).

### Apoptosis assay

An Annexin V-FITC/PI apoptosis detection kit (Cat#: 556,547; BD, USA) was used to measure cell apoptosis according to a previous study [35]. Approximately 1 × 10^5^ transfected OVCAR3 and SKOV3 cells were suspended in binding buffer containing 5 µL FITC and 10 µL PI and incubated in the dark for 20 min. Finally, the cells were suspended in binding buffer and subjected to FACSCanto II (BD, USA). The apoptosis rate was analyzed using FlowJo V7.6.1 (BD, USA).

### Wound healing assay

The wound healing assay was performed according to a previous study [36]. OVCAR3 and SKOV3 cells (1 × 10^6^) were cultured in 6-well plates. After cell monolayers were formed, we used a 10 μL sterile pipette to draw a straight line in the middle of the cells, and non-adherent cells were washed away. Then, the cells were incubated with serum-free medium for 24 h. Images of the scratch at 0 and 24 h were obtained using a light microscope.

### Transwell assay

The transwell assay was performed according to a previous study [37]. The lower transwell chamber (Cat#: 3422, Corning, USA) was prepared with a matrix gel (Corning, USA), and 10% FBS cell culture medium was added. The upper chamber was seeded with 8 × 10^4^ transfected OVCAR3 and SKOV3 cells without serum. After 48 h of incubation at 37°C, the invading cells were fixed with methanol for 20 min, and 0.1% crystal violet was used for staining at 25°C for 20 min. A light microscope was used to take the photographs.

### Luciferase assay

The luciferase assay was performed according to a previous study [14]. The predicted wild-type (WT) binding sequence of RHPN1-AS1 or TOP2A 3ʹ-UTR to miR-485-5p were amplified and inserted into psiCHECK2 vectors, which were named RHPN1-AS1 WT vectors or TOP2A 3ʹ-UTR WT vectors. The site-directed mutagenesis kit (SBS Genetech, China) was used to mutate the WT binding sequence of RHPN1-AS1 or TOP2A 3ʹ-UTR, and the produced mutant sequence was also inserted into psiCHECK2 vectors, which were named RHPN1-AS1 Mut1, RHPN1-AS1 Mut2, RHPN1-AS1 co-Mut, and TOP2A Mut vectors. The established vectors were transfected into OVCAR3 and SKOV3 cells treated with miR-485-5p mimic or NC for 48 h. Luciferase and Renilla activities were assessed using the Dual-Luciferase Reporter Assay System (Cat#: E1910, Promega, USA). The results were normalized to Renilla activity.

### RNA immunoprecipitation (RIP) assay

The interaction of lncRNA and miRNA in OVCAR3 and SKOV3 cells was determined using the EZ-Magna RIP kit (Cat#: #17-701, Sigma) according to a previous study [14]. The miR-485-5p mimic or NC and IgG (Cat#: ab172730, Abcam, UK) or Ago2 (Cat#: ab186733, Abcam) were incubated with the cell lysates for 2 h. Then, the solution was incubated with magnetic beads at 4°C overnight. Finally, RHPN1-AS1 was purified and analyzed using RT-qPCR.

### RNA-pull down assay

The RNA-pull down assay was performed according to a previous study [14]. Biotin-labeled miR-485-5p negative control (Bio-NC) and miR-485-5p (Bio-miR-485-5p) (Thermo) were used to treat OVCAR3 and SKOV3 cells for 48 h. Then, the cells were lysed and incubated with streptavidin beads (Cat#: #88,817, Thermo), overnight, at 4°C. RNeasy Mini Kit (Cat#: 74,104, QIAGEN, Germany) was used to purify the eluate from the beads, and RT-qPCR was used to detect TOP2A expression.

### Western blotting analysis

Protein expression was detected by western blot assay [24]. Transfected OVCAR3 and SKOV3 cell lysates were obtained using RIPA buffer (Cat#: #20-188, Sigma). After boiling at 95°C, the protein concentration was determined using a bicinchoninic acid Protein Assay kit (BioRad, USA). Then, 30 µg of protein was loaded on 10% SDS-PAGE at 72 V for 100 min and transferred to PVDF membranes at 72 V for 70 min. After blocking with 5% BSA (Sigma) in TBST containing 0.05% Tween-20 for 1 h at 25°C, anti-TOP2A (1:1,000, Cat#: ab219320, Abcam) and anti-GAPDH (1:2,000, Cat#: 5174, CST, USA) were used to incubate the membranes overnight at 4°C. Horseradish peroxidase-linked rabbit antibody (1:5,000, Cat#: ab6721, Abcam) was added, and the membranes were incubated for 1 h at 25°C. After three washes with TBST, protein bands were developed using an EasyBlot ECL kit (#C506668; Sangon, China). Relative TOP2A protein levels were normalized to that of GAPDH.

### Tumor xenograft

Tumor xenografts were used to assess tumor growth in vivo, according to a previous study [38]. For this, 4–6-week-old BALB/c nude mice (approximately 20 g) were purchased from the Wuhan University Center for Animal Experiment/Animal Biosafety Level III laboratory (ABSL-III lab) of Wuhan University (Wuhan, Hubei, China). Animal ethics approval was granted by the ethics committee of Wuhan Third Hospital. All mice were kept in a specific pathogen-free room with free access to water and food. 3 × 10^6^ SKOV3 cells transfected with sh-NC or sh-RHPN1-AS1 were administrated subcutaneously into each mouse. Tumor size was recorded every week. Five weeks later, the tumor weights were counted after euthanizing the mice.

### Statistical analysis

Data were analyzed using GraphPad Prism 8.0 (GraphPad Prism, USA) and presented as the mean ± SD from triplicate experiments. Differences between two or more groups were assessed by paired Student’s t-test or ANOVA with Dunnett’s or Tukey’s post hoc tests, respectively. Pearson correlation analysis was performed to analyze the relationship between RHPN1-AS1 and miR-485-5p or between TOP2A and miR-485-5p. Differences were considered statistically significant at *P* < 0.05.

## Results

In the current study, we ascertained the oncogenic role of RHPN1-AS1 *in vitro* and *in vivo*. We validated the assumption that the competing endogenous RNA (ceRNA) activity was manipulated by RHPN1-AS1 in OC. Our findings revealed a novel regulatory axis consisting of RHPN1-AS1/miR-485-5p/TOP2A. Our study may provide a therapeutic target for patients with OC.

### TOP2A and miR-485-5p were demonstrated to be downstream effectors of RHPN1-AS1 in OC

**Figure 1.**
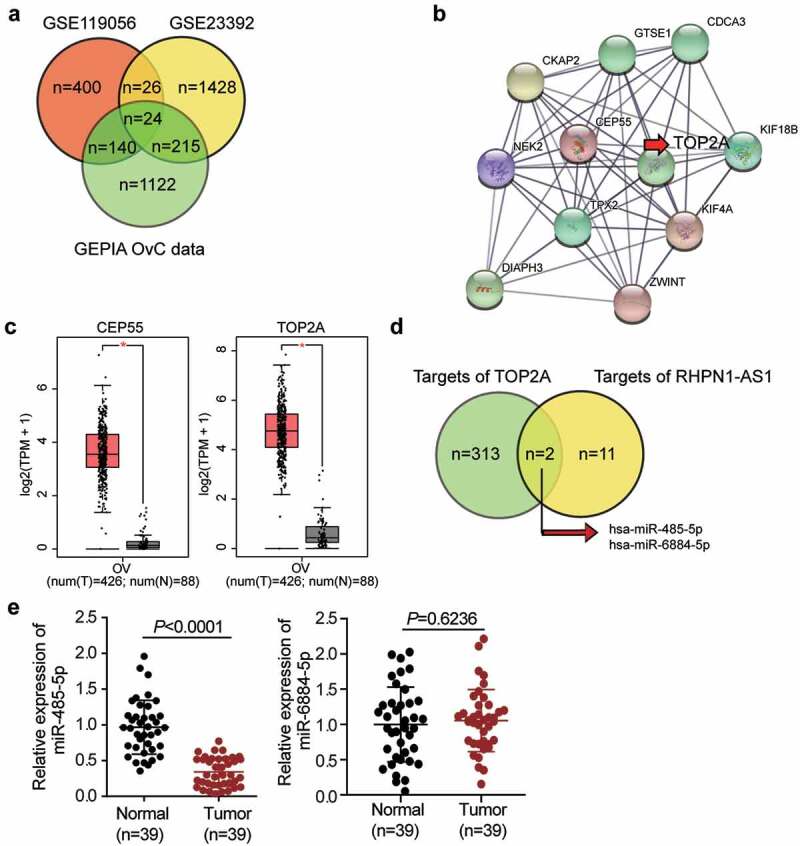
**TOP2A and miR-485-5p were selected as the downstream effectors of RHPN1-AS1 in OC**. (a) The identification of significantly upregulated genes in OC. The selection criteria for the three datasets were adjusted P < 0.01 and logFC≥1.5. (b) The overlapped genes were uploaded to STRING database for protein-protein interaction network analysis. (c) The expression of CEP55 and TOP2A in OC samples based on GEPIA database analysis. (d) The overlapped miRNAs that were predicted to interact with both RHPN1-AS1 and TOP2A mRNA. (e) Measurement of miR-485-5p and miR-6884-5p expression in OC tissues and normal tissues

RHPN1-AS1 has been reported to function as an OC promoter via a ceRNA network mechanism [12,13]. We analyzed two microarray datasets (GSE119056 and GSE23392) and obtained a list of differentially expressed genes in OC from the GEPIA database. Twenty-four overlapping genes were significantly upregulated in OC cells (Figure 1a and Supplementary Table 2). The results of our protein-protein interaction network analysis using the STRING database showed that 11 genes interacted closely with each other (Figure 1b). TOP2A and CEP55 interacted significantly within the network, and based on the data from GEPIA, TOP2A was more upregulated than CEP55 in tumor samples (Figure 1c). TOP2A has been reported to be a significant cancer progression-related gene [25,28,39–44]. However, there have been few reports of TOP2A being regulated by non-coding RNAs or being involved in the progression of OC. To identify an intermediate miRNA that interacts with both RHPN1-AS1 and *TOP2A* mRNA, we used TargetScan and starBase algorithms to predict the miRNAs targeting TOP2A and sponged by RHPN1-AS1, respectively. The results showed that miR-485-5p and miR-6884-5p might interact with both RHPN1-AS1 and *TOP2A* mRNA (Figure 1d). The miR-485-5p was downregulated in OC tissues in comparison with miR-6884-5p (Figure 1e), and it has been suggested to alleviate cisplatin resistance in OC cell lines [45] and participates in the ceRNA network in OC [21,46]. However, we hypothesized that miR-485-5p might be involved in a novel ceRNA network consisting of RHPN1-AS1 and TOP2A; thus, we conducted a series of experiments to test this hypothesis.

### RHPN1-AS1 silencing significantly inhibited the malignant proliferation

**Figure 2.**
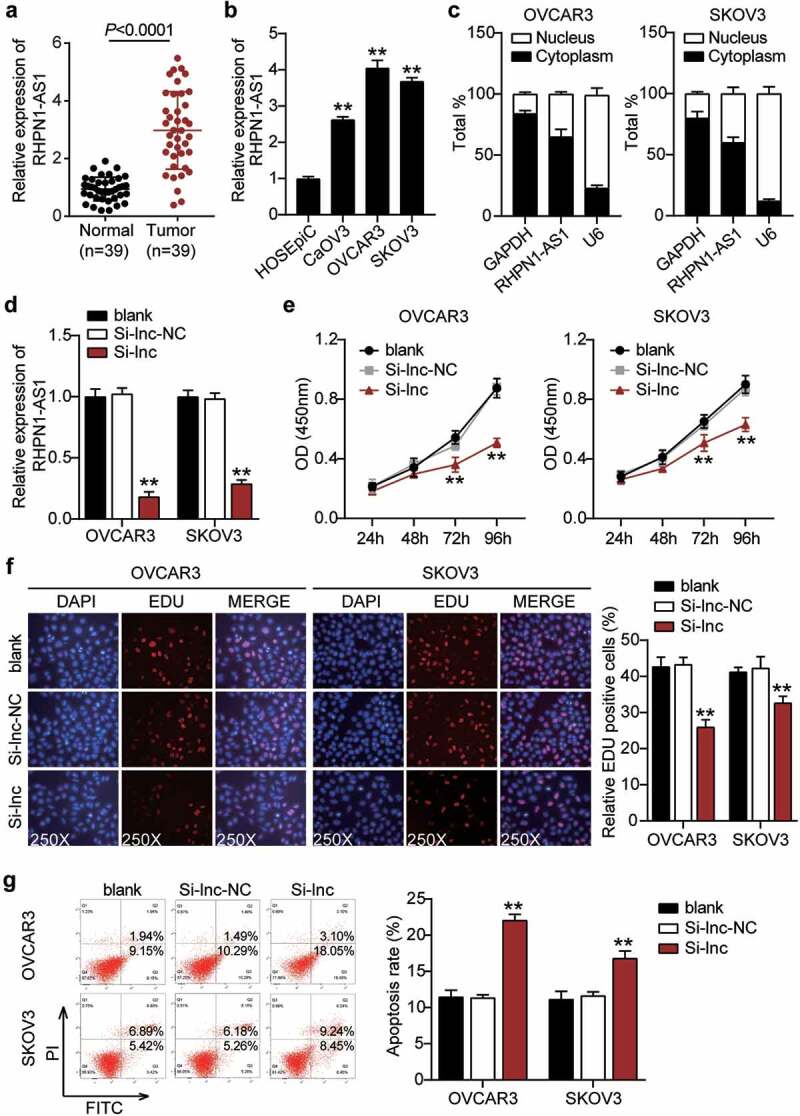
RHPN1-AS1 promoted cell growth, but inhibited cell apoptosis of OC cells

**Figure 3.**
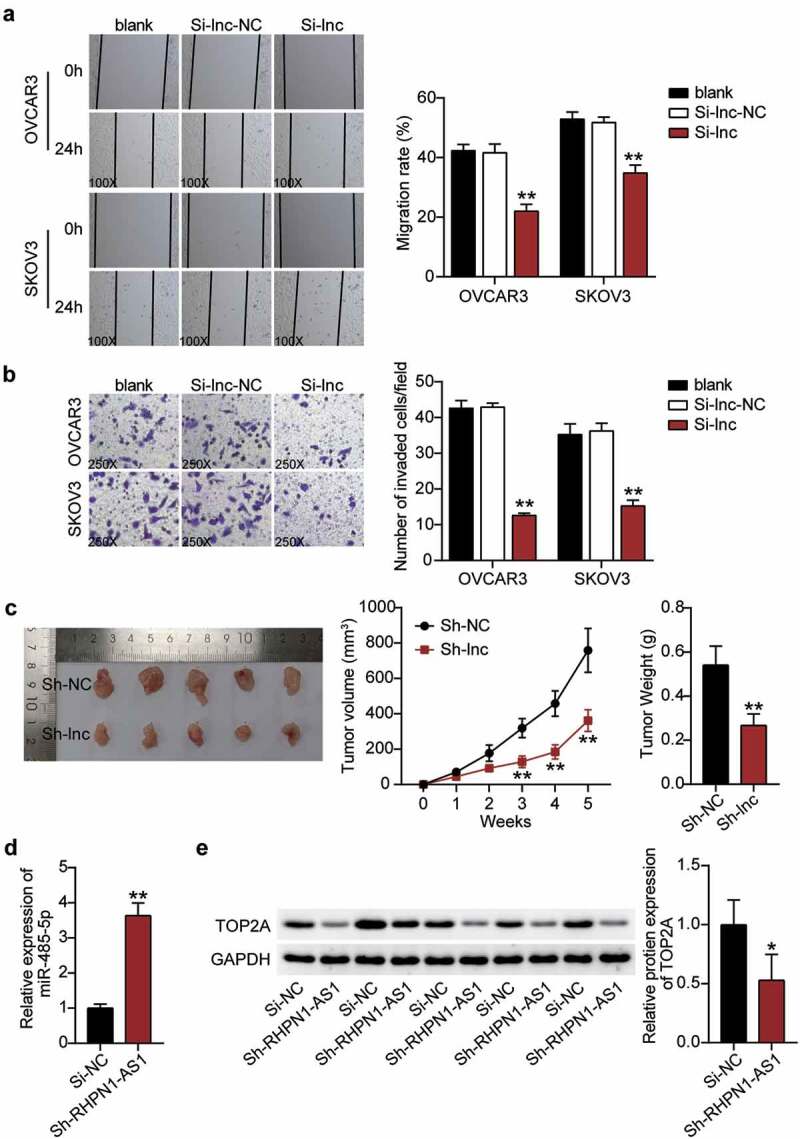
RHPN1-AS1 promoted cell migration and invasion of OC cells and retards tumor growth in vivo

After establishing the predicted ceRNA regulatory network of RHPN1-AS1/miR-485-5p/TOP2A, we further investigated whether RHPN1-AS1 participates in the progression of OC. First, we determined the RHPN1-AS1 expression in OC tissues and cells. OC tissues showed a 3-fold increase in RHPN1-AS1 expression compared to normal tissues (Figure 2a). All patients with OC were assigned into two groups (high RHPN1-AS1 group and low RHPN1-AS1 group) based on the median of RHPN1-AS1 expression. As shown in Table 1, the level of RHPN1-AS1 was strongly associated with FIGO stage, histological grade, and distant metastasis. However, no significant associations were found between RHPN1-AS1 expression and other clinical features, including age, tumor size, histological type, baseline neutrophils > 3.9, or chemotherapy regimens. The results from the above analysis suggest that tissue RHPN1-AS1 level is increased in OC and is associated with the progression of this malignant disease. The OC cells (CaOV3, OVCAR3, and SKOV3) showed higher RHPN1-AS1 expression levels than normal ovarian epithelial HOSEpiC cells, especially OVCAR3 and SKOV3 cells (Figure 2b). Next, we conducted a series of experiments on the two cells to study the RHPN1-AS1 function and found that the cytoplasm of both OVCAR3 and SKOV3 cells showed higher RHPN1-AS1 levels in contrast to the nucleus (Figure 2c). We further transfected siRNA-RHPN1-AS1 and NC into OVCAR3 and SKOV3 cells for functional detection. The Si-RHPN1-AS1 group showed 70% downregulated expression of RHPN1-AS1 in both OVCAR3 and SKOV3 cells compared to that in the control cells (Figure 2d). We found that the Si-RHPN1-AS1 groups showed significantly lower cell viability compared to control cells in both cell types (Figure 2e). Cell proliferation in the Si-RHPN1-AS1 group was 30% (OVCAR3) and 20% (SKOV3) lower than that in the control cells (figure 2f). Furthermore, the Si-RHPN1-AS1 groups displayed 2-fold (OVCAR3) and 1.5-fold (SKOV3) increased cell apoptosis compared to control cells (Figure 2g). Additionally, the Si-RHPN1-AS1 groups displayed 50% (OVCAR3) and 30% (SKOV3) decreased cell migration compared to control cells (Figure 3a). Moreover, the Si-RHPN1-AS1 group showed 75% (OVCAR3) and 50% (SKOV3) reduced cell invasion compared to the control cells (Figure 3b). SKOV3 cells transfected with sh-NC or sh-RHPN1-AS1 were subcutaneously injected into nude mice. The tumor volume and weight of the mice were recorded and plotted. As shown in Figure 3c, the volume and weight of tumors were reduced in nude mice administered RHPN1-AS1-silencing SKOV3 cells. As expected, loss of RHPN1-AS1 resulted in an increase in the expression of miR-485-5p and a decrease in the expression of TOP2A in tumor tissues from nude mice (Figures 3D and 3E). Taken together, the knockdown of RHPN1-AS1 dramatically restrained tumor growth *in vitro* and *in vivo*.

(A)Measurement of RHPN1-AS1 expression in OC tissues and normal tissues. (B) Measurement of RHPN1-AS1 expression in OC cell lines (CaOV3, OVCAR3, and SKOV3) and normal ovarian epithelial cells HOSEpiC. (C) The existence of GAPDH, RHPN1-AS1, and U6 in the cytoplasm and nucleus were detected in OVCAR3 and SKOV3 cell lines by RT-qPCR. (D) RT-qPCR analysis of RHPN1-AS1 in OVCAR3 and SKOV3 cells transfected with NC and Si-RHPN1-AS1. (E) Cell viability was detected in OVCAR3 and SKOV3 cells transfected with NC and Si-RHPN1-AS1 by CCK8 assay. (F) Cell proliferation was detected in OVCAR3 and SKOV3 cells transfected with NC and Si-RHPN1-AS1 by EdU assay. (G) Cell apoptosis was determined in OVCAR3 and SKOV3 cells transfected with NC and Si-RHPN1-AS1 by FITC apoptosis detection kit. **, P < 0.001 compared with HOSEpiC or blank. NC, negative control; Si-RHPN1-AS1, siRNA-RHPN1-AS1.

Cell migration ability was determined in OVCAR3 and SKOV3 cells transfected with NC and Si-RHPN1-AS1 by wound healing assay. (B) Cell invasion was determined in OVCAR3 and SKOV3 cells transfected with NC and Si-RHPN1-AS1 by transwell assay. (C) Tumor volume (left) and tumor weight (right) of SKOV3 cells transfected with sh-NC or sh-RHPN1-AS1 from nude mice with subcutaneous infection. (D) qRT-PCR detected miR-485-5p expression in tumors from nude mice. Western blot analysis of TOP2A expression in tumors from nude mice. *, P < 0.05; **, P < 0.001 compared with blank control or si-NC. NC, negative control; Si-RHPN1-AS1, siRNA-RHPN1-AS1.

### RHPN1-AS1 targeted to miR-485-5p

**Figure 4.**
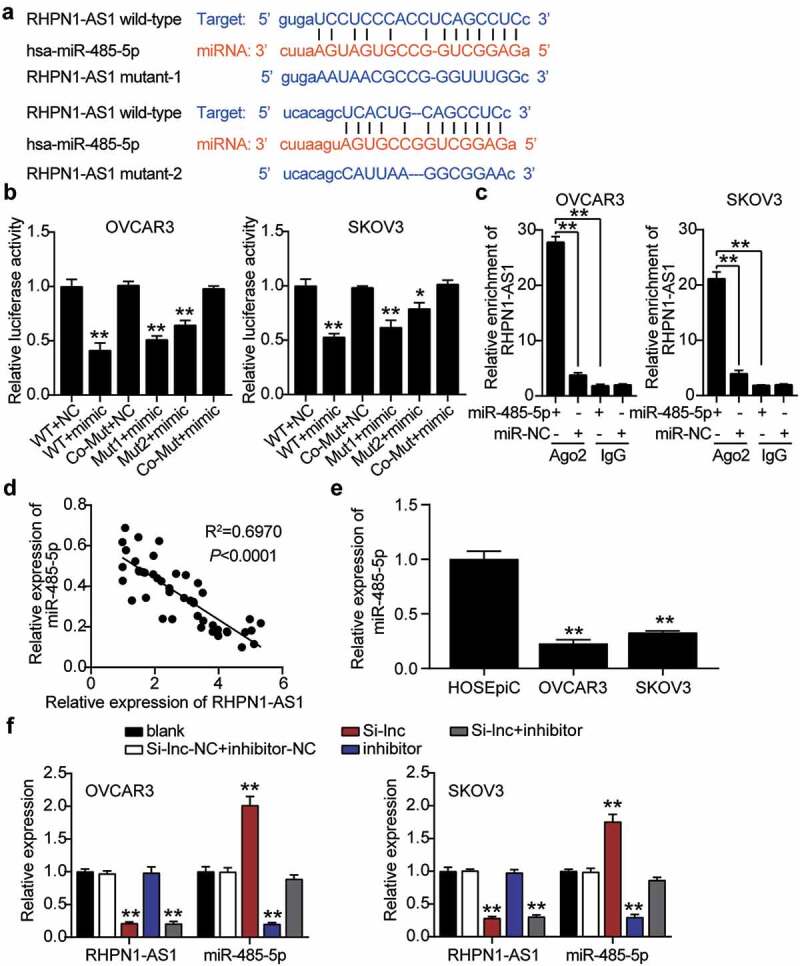
RHPN1-AS1 interacted with miR-485-5p in OC cells

Based on the above-mentioned predicted ceRNA network controlled by RHPN1-AS1, RHPN1-AS1 could sponge miR-485-5p. We further determined the interaction between miR-485-5p and RHPN1-AS1. To achieve this, we adopted a starBase analysis to disclose the target relationship between them. We found that miR-485-5p showed two binding site sequences with RHPN1-AS1 based on the prediction of starBase algorithms (Figure 4a). We co-treated miR-485-5p mimics and psiCHECK2 RHPN1-AS1-WT, Mut1, Mut2, or Co-Mut vectors into OVCAR3 and SKOV3 cells. The miR-485-5p mimics and co-psiCHECK2 RHPN1-AS1-WT showed nearly 60% downregulated luciferase activity compared to the NC group. Cells transfected with one of the psiCHECK2 RHPN1-AS1-Mut also showed decreased luciferase activity, whereas miR-485-5p mimics and psiCHECK2 RHPN1-AS1-Co-Mut showed no change in luciferase activity compared with the NC group, suggesting that miR-485-5p interacts with both sites of RHPN1-AS1 (Figure 4b). The RIP results also confirmed that, in both OVCAR3 and SKOV3 cells, RHPN1-AS1 interacted with miR-485-5p (Figure 4c). miR-485-5p levels reduced by 60% and were negatively correlated with RHPN1-AS1 levels in OC tissues (Figure 4d). The miR-485-5p content in both OVCAR3 and SKOV3 cells was over 50% lower than that in normal HOSEpiC cells (Figure 4e). We transfected miR-485-5p inhibitor, Si-RHPN1-AS1, and NC into OVCAR3 and SKOV3 cells to measure the regulation of RHPN1-AS1 in miR-485-5p. The Si-RHPN1-AS1 groups showed 2-fold enhanced miR-485-5p levels but 70% reduced RHPN1-AS1 levels compared to control cells. The miR-485-5p inhibitor groups showed a 70% reduction in miR-485-5p expression but the same RHPN1-AS1 expression compared with control cells. The Si-RHPN1-AS1+ inhibitor groups showed a 70% decrease in RHPN1-AS1 levels, but the miR-485-5p levels in the Si-RHPN1-AS1+ inhibitor groups were similar to those in the control cells (figure 4f).

StarBase analysis showed the predicted binding sequences of miR-485-5p for RHPN1-AS1. (B) Dual luciferase assay was performed in cells co-transfected with plasmids RHPN1-AS1-WT or RHPN1-AS1-MUT1 or MUT2 and NC mimics or miR-485-5p mimic in OVCAR3 and SKOV3 cells. (C) The enrichment of RHPN1-AS1 by treating with miR-485-5p mimic in OVCAR3 and SKOV3 cells through RIP assay. (D) Correlation analysis between miR-485-5p expression and RHPN1-AS1 expression. (E) RT-qPCR detection of miR-485-5p expression in OC cells (OVCAR3 and SKOV3 cells) and ovarian epithelial cells HOSEpiC. (F) RT-qPCR detection of miR-485-5p and RHPN1-AS1 expression in OVCAR3 and SKOV3 cells transfected with NC, Si-lnc, inhibitor, and Si-lnc+inhibitor. *, P < 0.05; **, P < 0.001 compared with blank. #, P < 0.05; ##, P < 0.001 compared with Si-RHPN1-AS1 + inhibitor. NC, negative control; WT, wild-type; MUT, Mutant; Si-lnc, SiRNA-RHPN1-AS1; inhibitor, miR-485-5p inhibitor; Si-lnc+inhibitor, SiRNA-RHPN1-AS1+ miR-485-5p inhibitor.

### Downregulation of miR-485-5p reversed the effect of silencing RHPN1-AS1 on the function of OC cells

**Figure 5.**
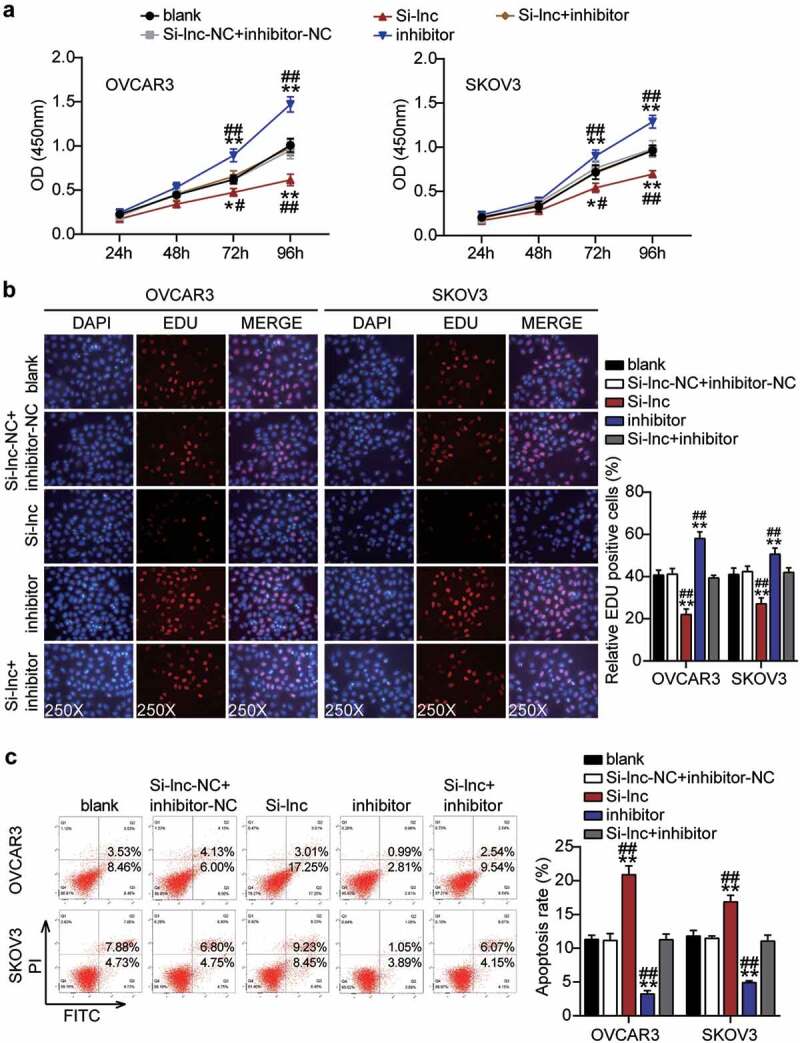
RHPN1-AS1 sponging miR-485-5p facilitated cell proliferation and repressed cell apoptosis of OC cells

**Figure 6.**
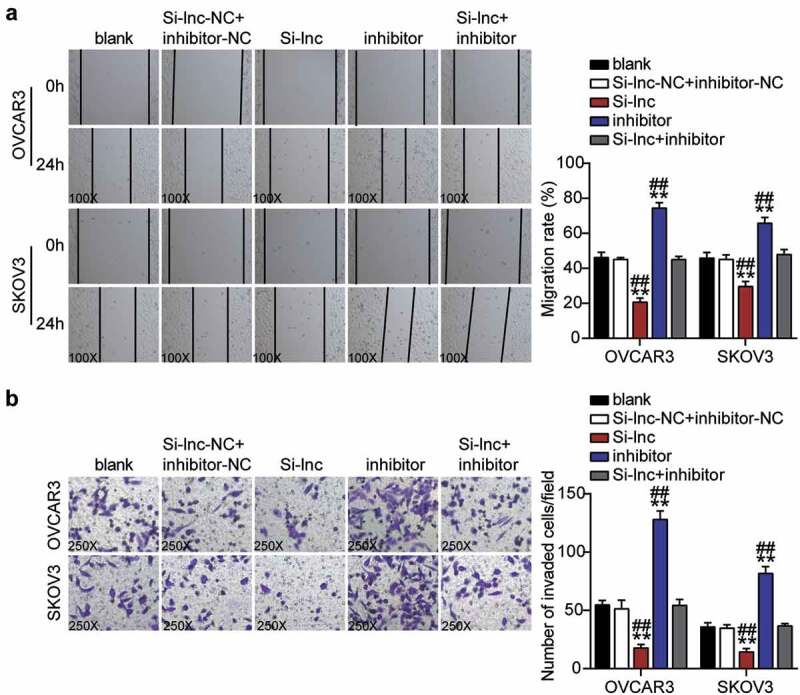
RHPN1-AS1 sponging miR-485-5p enhanced cell migration and invasion of OC cells

Since RHPN1-AS1 serves as an oncogenic lncRNA during OC malignancy, we further investigated whether miR-485-5p was necessary for RHPN1-AS1-mediated OC cell malignant phenotypes. We found that the inhibitor groups showed significantly enhanced cell viability compared to the control cells (Figure 5a). The inhibitor groups significantly increased cell proliferation by approximately 20% compared to that of the control cells (Figure 5b). Remarkably, the inhibitor groups inhibited more than 50% of cell apoptosis compared to the control cells (Figure 5c). The inhibitor groups dramatically enhanced approximately 50% and 30% cell migration compared with control cells in OVCAR3 and SKOV3, respectively (Figure 6a). In addition, the inhibitor groups also increased cell invasion by 50% compared to that in the control cells (Figure 6b). However, all the effects were counteracted by Si-RHPN1-AS1 in both OVCAR3 and SKOV3 cells, and RHPN1-AS1 inhibited the expression of miR-485-5p and hampered the malignant proliferation of OC cells.

Cell viability was detected in OVCAR3 and SKOV3 cells transfected with NC, Si-lnc, inhibitor, and Si-lnc+inhibitor. (B) Cell proliferation was detected in OVCAR3 and SKOV3 cells transfected with NC, Si-lnc, inhibitor, and Si-lnc+inhibitor. (D) Cell apoptosis was detected in OVCAR3 and SKOV3 cells transfected with NC, Si-lnc, inhibitor, and Si-lnc+inhibitor. *, P < 0.05; **, P < 0.001 compared with blank. #, P < 0.05; ##, P < 0.001 compared with Si-RHPN1-AS1 + inhibitor. NC, negative control; Si-lnc, SiRNA-RHPN1-AS1; inhibitor, miR-485-5p inhibitor; Si-lnc+inhibitor, SiRNA-RHPN1-AS1+ miR-485-5p inhibitor.

Cell migration was detected in OVCAR3 and SKOV3 cells transfected with NC, Si-lnc, inhibitor, and Si-lnc+inhibitor. (B) Cell invasion was detected in OVCAR3 and SKOV3 cells transfected with NC, Si-lnc, inhibitor, and Si-lnc+inhibitor. *, P < 0.05; **, P < 0.001 compared with blank. #, P < 0.05; ##, P < 0.001 compared with Si-RHPN1-AS1 + inhibitor. NC, negative control; Si-lnc, SiRNA-RHPN1-AS1; inhibitor, miR-485-5p inhibitor; Si-lnc+inhibitor, SiRNA-RHPN1-AS1+ miR-485-5p inhibitor.

### TOP2A was a downstream gene of miR-485-5p

**Figure 7.**
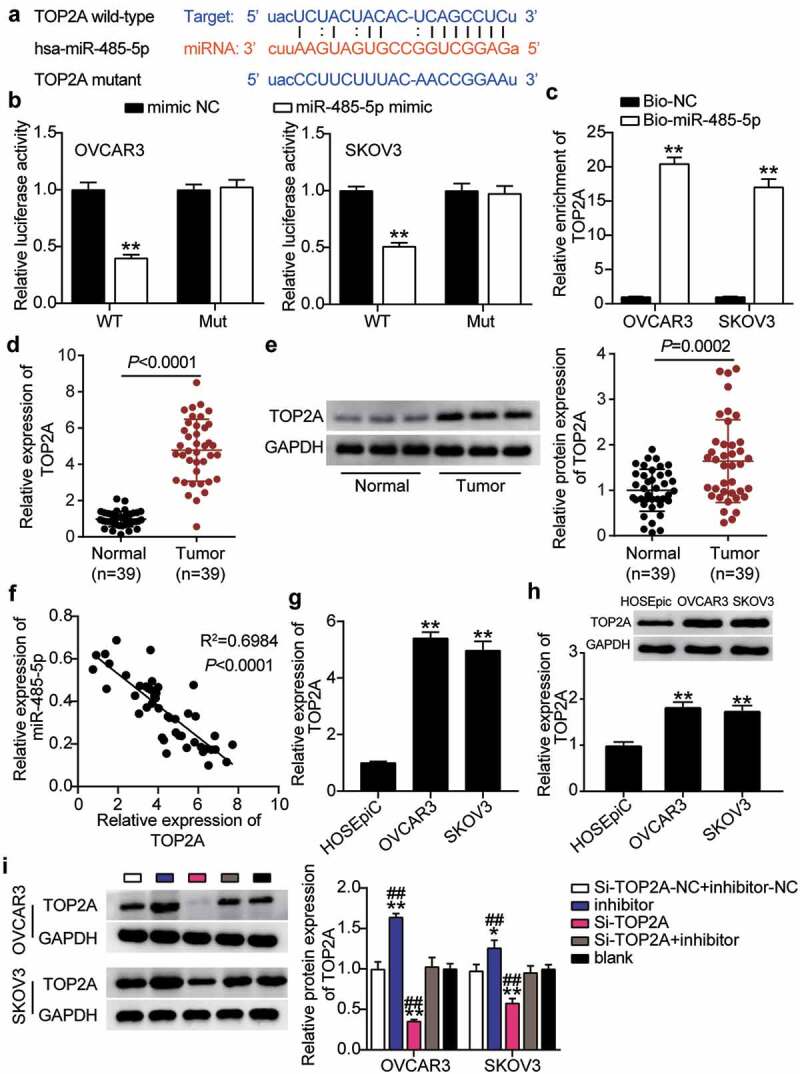
MiR-485-5p targeted to TOP2A and inhibited the expression of TOP2A in OC cells

To further verify the interaction between miR-485-5p and TOP2A, we first visualized the targeted binding using starBase (Figure 7a). OVCAR3 and SKOV3 cells co-treated with miR-485-5p mimics and psiCHECK2 TOP2A 3ʹ-UTR WT vectors showed nearly 50% decreased luciferase activity but showed no change in the psiCHECK2 TOP2A 3ʹ-UTR Mut vectors (Figure 7b). An RNA pull-down assay also revealed an interaction between miR-485-5p and TOP2A (Figure 7c). Furthermore, *TOP2A* mRNA and protein levels in OC tissues were significantly upregulated compared to normal tissues, whereas a negative correlation between miR-485-5p and TOP2A was observed in OC tissues (Figures 7D–7 F). The *TOP2A* mRNA and protein levels in OVCAR3 and SKOV3 cells were higher than those in normal HOSEpiC cells (Figure 7g and 7H). In addition, the western blot assay showed that TOP2A protein levels in OVCAR3 and SKOV3 cells decreased by 25% and 30%, respectively, after interference with RHPN1-AS1 (Supplementary Figure 1S). To clarify the function of the miR-485-5p-TOP2A axis in OC, we transfected siRNA-TOP2A, miR-485-5p inhibitor into OVCAR3 and SKOV3 cells. Here, the Si-TOP2A groups downregulated TOP2A protein levels by over 50%, whereas the inhibitor groups promoted TOP2A protein levels by nearly 1.5-fold compared to control cells. However, the Si+inhibitor groups showed the same levels as the control cells (Figure 7i).

StarBase showed the predicted binding sequences of TOP2A and miR-485-5p. (B) Dual luciferase assay was performed in cells co-transfected with plasmids TOP2A 3ʹUTR-WT or TOP2A 3ʹUTR-MUT and NC mimics or miR-485-5p mimic in OVCAR3 and SKOV3 cells. (C) The enrichment of RHPN1-AS1, miR-485-5p, and TOP2A by RNA pull-down assay. (D) Measurement of TOP2A mRNA expression in OC tissues and normal tissues. (E) Measurement of TOP2A protein expression in OC tissues and normal tissues. (F) The correlation between the relative expression level of miR-485-5p and TOP2A in OC tissues. (G) RT-qPCR detection of TOP2A expression in OC cells (OVCAR3 and SKOV3 cells) and ovarian epithelial cells HOSEpiC. (H) Western blotting detection of TOP2A expression in OC cells (OVCAR3 and SKOV3 cells) and ovarian epithelial cells HOSEpiC. (I) Measurement of TOP2A protein expression in OVCAR3 and SKOV3 cells transfected with NC, Si- TOP2A, inhibitor, and Si- TOP2A+inhibitor. *, P < 0.05; **, P < 0.001 compared with blank. #, P < 0.05; ##, P < 0.001 compared with Si-TOP2A+inhibitor. NC, negative control; WT, wild-type; MUT, Mutant; Si-TOP2A, SiRNA-TOP2A; inhibitor, miR-485-5p inhibitor; Si-TOP2A+inhibitor, SiRNA-TOP2A+ miR-485-5p inhibitor.

### TOP2A knockdown inhibited malignance proliferation and promoted apoptosis in OC cells and reversed the effect of interference with miR-485-5p

**Figure 8.**
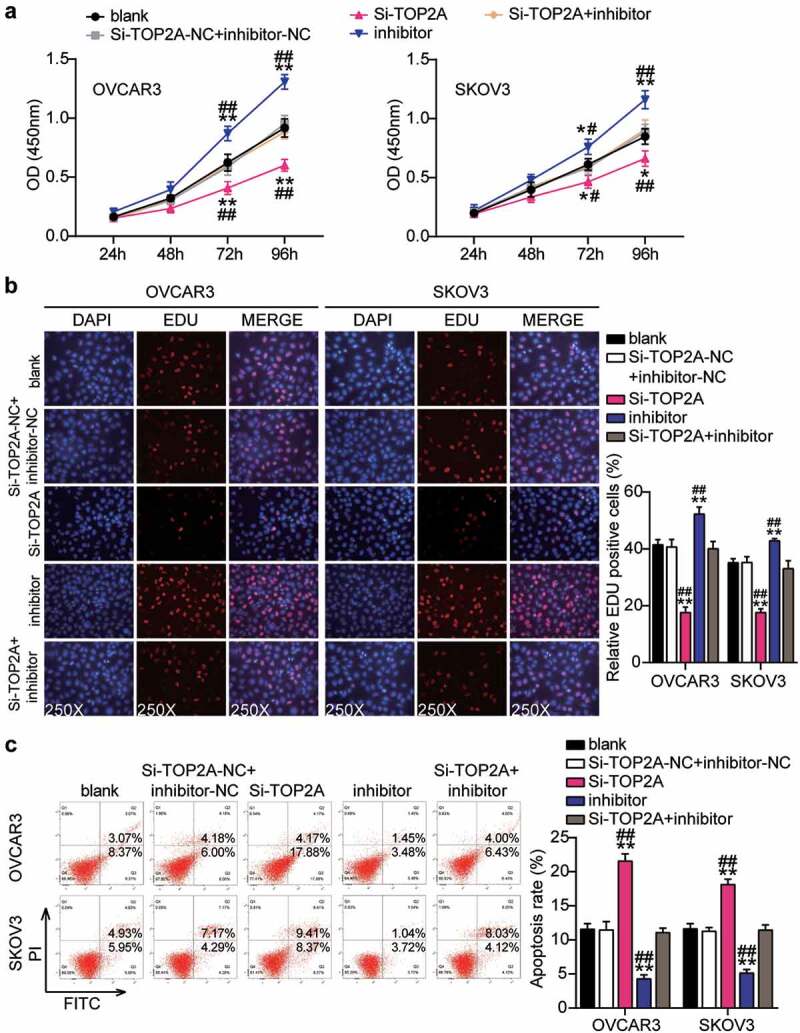
MiR-485-5p attenuated cell proliferation and elevated cell apoptosis by inhibiting TOP2A

**Figure 9.**
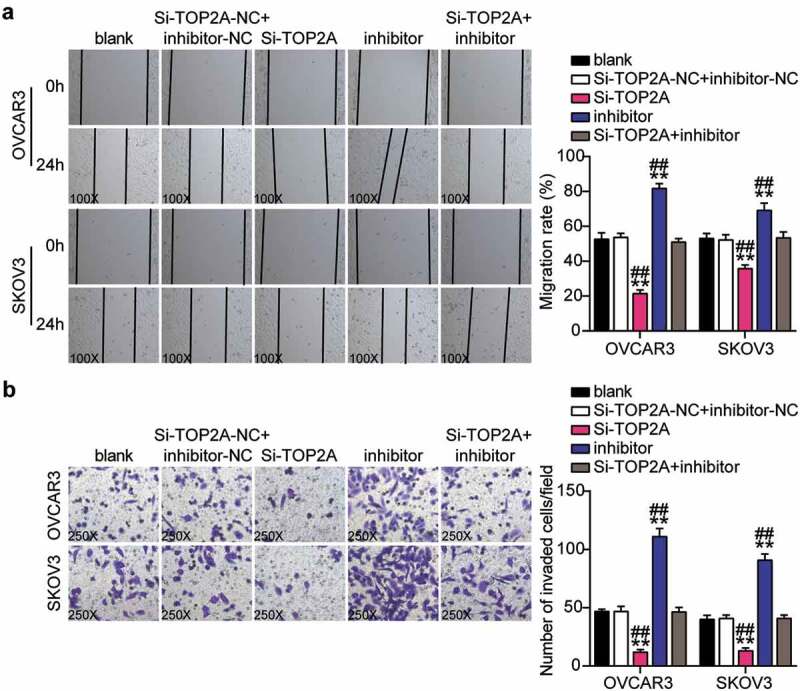
MiR-485-5p repressed cell migration and invasion by inhibiting TOP2A

Having demonstrated that miR-485-5p targets TOP2A, we investigated whether TOP2A was required for miR-485-5p function during OC malignancy. Therefore, a series of rescued assays were conducted to evaluate the biological function of the miR-485-5p-TOP2A axis in OC cells. The Si-TOP2A groups showed significantly reduced cell viability compared to the control cells (Figure 8a). At the same time, cell proliferation in the Si-TOP2A groups was repressed by approximately 50% compared to that in the control cells (Figure 8b). Moreover, the Si-TOP2A groups showed approximately 2-fold increase in cell apoptosis compared with control cells (Figure 8c). Additionally, the Si-TOP2A groups reduced cell migration by 60% (OVCAR3) and 25% (SKOV3) compared with control cells (Figure 9a). Meanwhile, the Si-TOP2A groups showed approximately 70% cell invasion compared with control cells in both cell lines (Figure 9b). However, these effects were prevented by treatment with the Si-TOP2A+inhibitor in both OVCAR3 and SKOV3 cells. Thus, TOP2A knockdown suppressed the proliferation, migration, and invasion of OC cells and facilitated apoptosis, which eliminated the effect of the miR-485-5p interference.

Cell viability was detected in OVCAR3 and SKOV3 cells transfected with NC, Si-TOP2A, inhibitor, and Si-TOP2A+inhibitor. (B) Cell proliferation was detected in OVCAR3 and SKOV3 cells transfected with NC, Si-TOP2A, inhibitor, and Si-TOP2A+inhibitor. (C) Cell apoptosis was detected in OVCAR3 and SKOV3 cells transfected with NC, Si-TOP2A, inhibitor, and Si-TOP2A+inhibitor. *, P < 0.05; **, P < 0.001 compared with blank. #, P < 0.05; ##, P < 0.001 compared with Si-TOP2A+inhibitor. NC, negative control; Si-TOP2A, SiRNA-TOP2A; inhibitor, miR-485-5p inhibitor; Si-TOP2A+inhibitor, SiRNA-TOP2A+ miR-485-5p inhibitor.

(A) Cell migration was detected in OVCAR3 and SKOV3 cells transfected with NC, Si-TOP2A, inhibitor, and Si-TOP2A+inhibitor. (B) Cell invasion was detected in OVCAR3 and SKOV3 cells transfected with NC, Si-TOP2A, inhibitor, and Si-TOP2A+inhibitor. *, P < 0.05; **, P < 0.001 compared with blank. #, P < 0.05; ##, P < 0.001 compared with Si-TOP2A+inhibitor. NC, negative control; Si-TOP2A, SiRNA-TOP2A; inhibitor, miR-485-5p inhibitor; Si-TOP2A+inhibitor, SiRNA-TOP2A+ miR-485-5p inhibitor.

## Discussion

Recent investigations have revealed the oncogenic function of RHPN1-AS1 interacting with miR-596 during OC progression. However, the underlying mechanism of the functional influence of RHPN1-AS1 on miRNA-mediated oncogenes has not been fully explored. Our study found that RHPN1-AS1 suppressed miR-485-5p levels, which further accelerated cell survival and reduced apoptosis by upregulating TOP2A in OC cells.

The lncRNA RHPN1-AS1 was previously reported to be upregulated in various cancers and is frequently associated with cancer pathophysiology [6,10,47]. In OC, robust lncRNA RHPN1-AS1 expression was detected in cancer tissues; it serves as an unfavorable prognosticator and promotes tumor cell proliferation and metastasis [14] Our study showed that RHPN1-AS1 expression was enhanced in OC tissues and cells. Downregulation of RHPN1-AS1 significantly reduced the survival of OVCAR3 and SKOV3 cells and increased apoptosis. Furthermore, RHPN1-AS1 silencing suppressed ovarian tumorigenesis. Our findings provide evidence for further clarification of the carcinogenic role of RHPN1-AS1 in ovarian carcinomas.

The lncRNA are known to interact with sponges for various miRNAs, thereby regulating multiple cellular functions through a complex network of interactions. Thus, targeting lncRNAs may interfere with cancer progression. Currently, RHPN1-AS1 is known to exhibit ceRNA activity against several antitumor miRNAs, such as miRNA-129, miR-625, miR-7-5p, and miR-485-5p, contributing to cancer tumorigenesis and progression [14,48–50]. Based on our bioinformatics prediction, we found that miR-485-5p is a novel target of RHPN1-AS. There is extensive literature supporting the suppressor function of miR-485-5p in the pathogenesis of different cancers, especially in OC [18,51]. Yang *et al*. reported that miR-485-5p levels were remarkably reduced in OC tissues and cells and that the upregulation of miR-485-5p dramatically suppressed SKOV3 cell proliferation, migration, and invasion, and induced cycle arrest and apoptosis *in vivo* and *in vitro* [20]. Notably, miR-485-5p was downstream of the lncRNA LINC01224, and the knockdown of LINC01224 attenuated OC cell growth; however, the effects were counteracted by miR-485-5p inhibitor [21]. Similarly, we found that miR-485-5p levels were downregulated in OC tissues and cells. Furthermore, targeting analysis validated that RHPN1-AS1 directly interacts with miR-485-5p. More importantly, interference with miR-485-5p accelerated cell growth and reduced apoptosis in OC cells. RHPN1-AS1 reduced miR-485-5p levels and promoted the evolvement of OC, revealing that RHPN1-AS1 relieved the suppression of miR-485-5p sponge, thereby promoting cancer progression. The negative trend between RHPN1-AS1 and miR-485-5p also supported the finding that RHPN1-AS1 targeted miR-485-5p. The miR-485-5p is involved in the regulation of oxidative stress and thereby plays a critical role in cardiovascular and cerebrovascular diseases [52,53]. Of note, oxidative stress is well documented as an inducer of cancer onset and development. Indeed, the involvement of non-coding RNAs cannot be neglected in oxidative stress-induced cancer initiation and development [54]. In future work, we will focus on the effect of the RHPN1-AS1/ miR-485-5p axis on oxidative stress during OC malignancy.

We found, for the first time, that miR-485-5p could bind to the 3ʹ-UTR of TOP2A and had a direct regulatory role in TOP2A expression. TOP2A is a DNA topoisomerase involved in DNA replication and transcription. TOP2A functions as an oncogene in various cancers, including OC [24,55,56]. TOP2A silencing reduces the phosphorylation of Smad2 and Smad3 and impairs the malignant characteristics of cancer cells, which is implicated in the carcinogenesis of high-grade serous OC [28]. Eleonora et al. pointed out that TOP2A expression levels predicted the efficacy of PEGylated lysosomal doxorubicin against epithelial OCs [29]. However, the functional impact of lncRNA-miRNA interactions on TOP2A in OC has not been explored previously. Herein, we further elucidated the role of TOP2A in OC and showed that TOP2A expression was remarkably upregulated in OC tissues and cells. Consistent with previous investigations, we found that the knockdown of TOP2A suppressed cell growth but enhanced apoptosis in OC cells. Moreover, targeting assays demonstrated that miR-485-5p exerts its regulatory effect on TOP2A through complementary binding. TOP2A-silence hinders the malignant behavior of OC cells, and this can be restored by the miR-485-5p inhibitor. More importantly, silencing RHPN1-AS1 led to an obvious increase in the expression of TOP2A in nude mice. All data suggest that RHPN1-AS1 competes with TOP2A for miR-485-5p and reduces the suppression of miR-485-5p on TOP2A degradation, thereby participating in the initiation and progression of OC.

TOP2A regulates various signaling pathways and participates in the evolvement of cancer [24,26,28]. Pei et al. reported that TOP2A induces pancreatic cancer progression by boosting β-catenin signaling [24]. Wang et al. also reported that TOP2A elevated cell growth by activating the PI3K/AKT signaling pathway in cervical cancer [26]. Notably, TOP2A promoted cell proliferation, migration, and invasion by activating the TGF-β/Smad pathway in OC cells [28]. Therefore, the signaling pathways that participate in both RHPN1-AS1 and miR-485-5p in OC require further examination.

## Conclusion

Taken together, these findings revealed that RHPN1-AS1 sponging miR-485-5p accelerated the progression of OC by elevating TOP2A expression, which is a promising target for the treatment of OC.

## Supplementary Material

Supplemental Material

## Data Availability

The datasets used and/or analyzed during the current study are available from the corresponding author on reasonable request wang_gf69@163.com

## Highlights

- Robust expression of RHPN1-AS1 is related to the clinical characters of OC patients.
- RHPN1-AS1 silencing significantly inhibited the malignant proliferation.
- RHPN1-AS1 sponges miR-485-5p and elevates TOP2A expression.
