# Supplementary material for: Rhophilin rho GTPase binding protein 1-antisense RNA 1 (RHPN1-AS1) promotes ovarian carcinogenesis by sponging microRNA-485-5p and releasing DNA topoisomerase II alpha (TOP2A)
Source: Bioengineered. 2021 Dec 7;12(2):12003–22. doi: 10.1080/21655979.2021.2002494 (PMC8810118; doi:10.1080/21655979.2021.2002494)
Supplement: Supplemental Material [file KBIE_A_2002494_SM8520.zip › supplementary/Supplementary legend.docx]

**Supplementary Figure 1.** The expression of TOP2A protein in OVCAR3 and SKOV3 cells transfected with siRNA-RHPN1-AS1. NC, negative control; Si-lnc, siRNA-RHPN1-AS1. ^**^, *P*< 0.001 compared with blank.
