# Supplementary material for: Rhophilin rho GTPase binding protein 1-antisense RNA 1 (RHPN1-AS1) promotes ovarian carcinogenesis by sponging microRNA-485-5p and releasing DNA topoisomerase II alpha (TOP2A)
Source: Bioengineered. 2021 Dec 7;12(2):12003–22. doi: 10.1080/21655979.2021.2002494 (PMC8810118; doi:10.1080/21655979.2021.2002494)
Supplement: Supplemental Material [file KBIE_A_2002494_SM8520.zip › supplementary/Supplementary_Table_1.docx]

Table 1 Associations of tissue RHPN1-AS1 clinicopathological characteristics in 39 ovarian cancer samples.

| **Characteristic** | N=39 | RHPN1-AS1 expression | | P value |
| --- | --- | --- | --- | --- |
|  |  | High n=20 | Low n=19 |  |
| **Age (years)** |  |  |  | 0.527 |
| ≤ 56 | 18 | 8 | 10 |  |
| > 56 | 21 | 12 | 9 |  |
| **Tumor Size (cm)** |  |  |  | 0.200 |
| ≥ 2 | 23 | 14 | 9 |  |
| < 2 | 16 | 6 | 10 |  |
| **FIGO stage** |  |  |  | 0.008* |
| 1 | 6 | 1 | 5 |  |
| 2 | 7 | 1 | 6 |  |
| 3 | 22 | 14 | 8 |  |
| 4 | 4 | 4 | 0 |  |
| **Baseline neutrophils > 3.9** |  |  |  | 0.092 |
| Yes | 33 | 19 | 14 |  |
| No | 6 | 1 | 5 |  |
| **Histological grade** |  |  |  | 0.001* |
| 1 | 13 | 1 | 12 |  |
| 2 | 18 | 13 | 5 |  |
| 3 | 8 | 6 | 2 |  |
| **Histological type** |  |  |  | 0.105 |
| Serous | 24 | 15 | 9 |  |
| Non-serous | 15 | 5 | 10 |  |
| **Distant metastasis** |  |  |  | 0.010* |
| Yes | 21 | 15 | 6 |  |
| No | 18 | 5 | 13 |  |
| **Chemotherapy regimens** |  |  |  | 0.476 |
| Carbo + Tax | 25 | 11 | 14 |  |
| Carbo monotherapy | 11 | 7 | 4 |  |
| Carbo + Other | 3 | 2 | 1 |  |

FIGO, International Federation of Gynaecology and Obstetrics; Carbo, Carboplatin; Tax, Taxol (Paclitaxel). *, P<0.05 using Fisher exact test.
