# Supplementary material for: Rhophilin rho GTPase binding protein 1-antisense RNA 1 (RHPN1-AS1) promotes ovarian carcinogenesis by sponging microRNA-485-5p and releasing DNA topoisomerase II alpha (TOP2A)
Source: Bioengineered. 2021 Dec 7;12(2):12003–22. doi: 10.1080/21655979.2021.2002494 (PMC8810118; doi:10.1080/21655979.2021.2002494)
Supplement: Supplemental Material [file KBIE_A_2002494_SM8520.zip › supplementary/Supplementary_table_2.docx]

Supplementary table 2 The 24 overlapped genes from GSE119056, GSE23392 and GEPIA

| Gene Symbol | GSE119056 | | GSE23392 | | | GEPIA | |
| --- | --- | --- | --- | --- | --- | --- | --- |
|  | log_2_FC | adj.P value | | log_2_FC | adj.P value | log_2_FC | adj.P value |
| DIAPH3 | 4.119 | 1.74E-03 | | 4.219 | 7.51E-03 | 1.583 | 1.90E-69 |
| PITX1 | 5.038 | 1.82E-03 | | 3.298 | 6.20E-03 | 2.48 | 1.35E-23 |
| ICOSLG | 3.346 | 1.84E-03 | | 4.183 | 2.61E-03 | 1.55 | 4.60E-48 |
| ADGRG1 | 4.571 | 2.78E-03 | | 3.628 | 3.62E-03 | 3.804 | 8.20E-101 |
| CELSR1 | 3.512 | 3.04E-03 | | 5.203 | 6.76E-03 | 3.651 | 1.08E-117 |
| TOP2A | 5.239 | 3.05E-03 | | 4.110 | 8.88E-03 | 4.32 | 4.16E-139 |
| CKAP2 | 2.702 | 4.03E-03 | | 3.813 | 8.74E-03 | 2.062 | 5.09E-84 |
| CEP55 | 5.469 | 5.76E-03 | | 3.339 | 2.57E-03 | 3.412 | 1.45E-125 |
| HES6 | 3.459 | 6.29E-03 | | 3.463 | 9.60E-03 | 1.74 | 1.58E-35 |
| TPX2 | 5.466 | 6.39E-03 | | 3.499 | 8.54E-03 | 4.732 | 1.76E-163 |
| KIF4A | 1.951 | 6.50E-03 | | 5.200 | 1.90E-03 | 2.363 | 7.19E-91 |
| SDC3 | 2.758 | 6.67E-03 | | 4.449 | 7.08E-03 | 2.148 | 8.15E-43 |
| SYNDIG1 | 3.192 | 7.26E-03 | | 3.468 | 6.50E-03 | 2.528 | 2.01E-37 |
| SYK | 4.158 | 7.30E-03 | | 3.924 | 9.48E-03 | 3.345 | 8.95E-110 |
| CDCA3 | 2.884 | 7.39E-03 | | 4.112 | 4.11E-03 | 2.323 | 4.22E-71 |
| GTSE1 | 3.584 | 7.44E-03 | | 6.695 | 6.37E-04 | 2.119 | 3.66E-72 |
| STK17B | 2.807 | 7.47E-03 | | 5.453 | 2.93E-03 | 1.807 | 2.43E-53 |
| ZWINT | 4.344 | 7.77E-03 | | 3.670 | 2.80E-03 | 3.063 | 1.70E-124 |
| GIPC1 | 1.696 | 8.03E-03 | | 3.949 | 9.11E-03 | 1.759 | 2.67E-66 |
| KIF18B | 2.339 | 8.24E-03 | | 3.128 | 4.76E-03 | 2.776 | 1.11E-93 |
| STC2 | 4.910 | 8.32E-03 | | 5.616 | 2.02E-03 | 2.461 | 1.56E-54 |
| NEK2 | 5.243 | 8.36E-03 | | 3.457 | 3.66E-03 | 3.426 | 1.14E-129 |
| CCDC88C | 2.602 | 8.46E-03 | | 3.683 | 5.44E-03 | 1.876 | 7.09E-52 |
| HKDC1 | 1.593 | 9.33E-03 | | 3.129 | 4.58E-03 | 1.925 | 9.02E-50 |
